# Charles W. McCall, D.D.S.

**Published:** 1900-08

**Authors:** 


### CHARLES W. McCALL, D.D.S.

Dr. McCall died June 7, 1900, at Binghamton, N. Y., in his fiftieth year.

Dr. McCall was born in Franklin, Delaware County, N. Y., on August 24, 1850. He was a son of the late Dr. S. H. McCall, for many years one of the most prominent dentists of Binghamton and the State of New York.

Dr. Charles W. McCall was most carefully prepared for the practise of his profession, first as a student in his father's office, afterwards in laboratories and offices in New York, graduating from the New York College of Dentistry in the Class of 1876. He practised for a short time in South Orange, N. J., when he returned to Binghamton to associate himself in practice with his father. After the death of his father he continued the practice, removing it to his own residence at 82 Chenango Street, where he maintained one of the most lucrative practices in this part of the State until his death, which occurred after only one week's illness.

Dr. McCall was one of the most prominent and active members of the Sixth District Dental Society, but so free from selfishness that he much preferred to see its offices in the hands of others rather than his own, but was twice its Vice-President, twice its President, and a member of its Board of Censors at the time of his death.

At the celebration of the twenty-fifth anniversary of the Society he, the son of its first President, was very fitly the presiding officer.

He was a member of the First Presbyterian Church, and prominent in business and social circles in Binghamton.

Since the organization of the Board of Trustees of the Barlow School of Industrial Arts at Binghamton he was a member of that body, also a popular and prominent member of the Dobson Club and the Broome County Country Club.

It is difficult to convey to those who did not know him a proper idea of Dr. McCall, genial, unselfish, sympathetic, ready to listen to the joys or sorrows of all who came to him, as his clergyman said, "Sorrowing with those who sorrowed, rejoicing with those who rejoiced." It did seem when you told him of some good fortune that had befallen you that it gave him more delight than if it were his own; and when grief or misfortune had come to one, he always seemed able to pick out the blessing attendant upon it and make it to you the most prominent feature.

Fond of languages, he found time from a large practice to pursue their study. Of broad culture and artistic tastes, his home was not only a delight to him but to all his friends and visitors. Popular as he was in social and club life, and fond of foreign travel, having travelled quite extensively abroad, yet his devotion was to his home, where he led an ideal life with his wife and son, both of whom were his most frequent companions when the day's work was ended.

Industrious, business-like, yet professionally ethical, always with words of praise for his competitors, dentistry has sustained a loss in his death, his friends and the Sixth District Dental Society of the State of New York an irreparable one.

He was married to Miss Elizabeth Lyon Mandeville April 7, 1880, and is survived by her and one son, John Oppie McCall, a member of the Senior Class of Yale College.

